# 

**Published:** 1897-12-04

**Authors:** 


					THE HOSPITAL. ABSOLUTELY PURE, therefore BEST. Dec. 4tii, 1897.
CADBURY'S war CADBURY'S
OOCOA rip i|=f Mr* COOOA
B> mm NO ALKALIES USED.
EH
'RWICH HOSPITAL
No. 584. Vol. XXIII. [TS;Jp?] Saturday,
Dec. 4th, 1897.
3it56.ermB'l PRICE TWOPENCE.
L. I ? ( . t I I

				

